# Sweetpotato Leaves Inhibit Lipopolysaccharide-Induced Inflammation in RAW 264.7 Macrophages via Suppression of NF-κB Signaling Pathway

**DOI:** 10.3390/foods10092051

**Published:** 2021-08-31

**Authors:** Hyun-Dong Cho, Cindi Brownmiller, Harun Sorker, Shahidul Islam, Sun-Ok Lee

**Affiliations:** 1 Department of Food Science, University of Arkansas, Fayetteville, AR 72704, USA; chd0811@hanmail.net (H.-D.C.); cbrownm@uark.edu (C.B.); 2 Department of Agriculture, University of Arkansas, Pine Bluff, AR 71601, USA; Harun_rashid007@yahoo.com; 3 Department of Horticulture, University of Arkansas, Pine Bluff, AR 71601, USA; islams@uapb.edu

**Keywords:** anti-inflammation, NF-κB signaling pathway, phenolic compounds, sweetpotato leaves

## Abstract

Limited information is available regarding the health-promoting activities of sweetpotato leaves (SPL). The present study investigated antioxidant and anti-inflammatory effects, and phenolic contents in 29 SPL cultivars harvested in 2018 and 2019. Extracts showed total phenolic contents 9.4–23.1 mg gallic acid equivalent/g, and DPPH radical scavenging activity indicated 36.6–247.3 mM of Trolox equivalent/g. SPL extracts were identified to contain bioactive components such as, chlorogenic acid (11.7–22.1 μg/mg), 3,4-dicaffeoylquinic acid (16.3–59.9 μg/mg), 3,5-dicaffeoylquinic acid (50.9–72.7 μg/mg), chlorophyll B (6.1–12.3 μg/mg), lutein (1.9–4.9 μg/mg), chlorophyll A (2.7–4.3 μg/mg) and β-carotene (0.1 ≤ μg/mg). RAW 264.7 murine macrophage cells were pretreated with 100–200 μg/mL of SPL extracts and 20 μM of dexamethasone, and inflammation was stimulated by lipopolysaccharide (LPS, 100 ng/mL) treatment for 24 h. In LPS-treated cells, prostaglandin E2 production and COX-2 expression were not downregulated by pretreatment of SPL extracts. However, SPL pretreated cells showed significant suppression of nitric oxide (NO), TNF-α, and IL-1β levels under the LPS-induced inflammatory condition. In addition, SPL extracts induced an anti-inflammatory effect in LPS-stimulated RAW 264.7 cells through suppression of NF-κB nuclear translocation, IKK-α and IκB-α phosphorylation, and iNOS expression. These results indicate that SPL extract can be utilized as a functional food ingredient.

## 1. Introduction

Over the past few years, numerous studies have reported that inflammation response is related to the incidence of chronic diseases, such as obesity, cancer, diabetes, and Alzheimer’s disease [1,2,3,4]. A recent study has indicated that a hyperinflammatory response induced by SARS-CoV-2 is a major reason for an increase in the death rate in infected people [5]. Not only in these pathological conditions but also acute illnesses including systemic inflammatory response syndrome, sepsis, and septic shock, immune systems play a critical role to produce and release pro-inflammatory molecules, cytokines, and chemokines [6]. Although proper induction of an acute inflammatory system can be helpful to prevent infection and tissue repair, the excessive inflammatory response has to be down-regulated via continuous management and therapeutic intervention.

Several inflammation-related chronic diseases including obesity, cancer, diabetes, and Alzheimer’s disease are necessarily related to an increase in pro-inflammatory effectors including nitric oxide (NO) and various pro-inflammatory cytokines containing tumor necrosis factor α (TNF-α), interleukin-6 (IL-6), and interleukin-1β (IL-1β) [7]. The nuclear factor-κB (NF-κB) family is one of the most potent pro-inflammatory factors involved in the expression of numerous transcription factors in various steps of the immune and inflammation process [8]. The NF-κB protein family is normally expressed and stably resides in the cytoplasm with polymerization of the inhibitor-κB (IκB) protein family which inhibits NF-κB activation. However, under the inflammatory condition, IκB proteins are phosphorylated and degraded through site-specific phosphorylation by IκB kinases (IKK). After ubiquitin-dependent degradation of IκBα, remaining NF-κB subunits translocate into the nucleus to mediate inflammatory responses [8,9]. Translocated NF-κB subunits bind to various transcription sites and affect the expression of inflammation-related cytokines, chemokines, adhesion molecules, anti-apoptotic factors, and enzymes [10]. Hence, suppression of disproportionate inflammation targeting the NF-κB signaling pathway can be used as an efficient strategy to prevent inflammation-related chronic diseases.

Since some studies have focused on the biological functionality of food materials to prevent and treat chronic diseases, the interest in functional food ingredients and/or phenolic compounds has been steadily growing worldwide [11]. Sweetpotato (*Ipomoea batatas* L.), a dicotyledonous plant that belongs to the morning glory family and Convolvulaceae, is one of the most popular food crops to contribute to the human diet, animal feeding, and food-industrial bioprocessing sources in the world [12]. It is composed of tuber, stem, and leaf parts, and the sweetpotato leaves (SPL) also have been consumed as a food material and for traditional medicine in East Asian countries, such as Taiwan, Japan, and China [13]. However, still many countries still have a strong perception of SPL as a byproduct of the food industry, and its value of functionality has been ignored. Although the nutritional quality of SPL varies according to cultivar, harvest period, soil, and processing method, it has been reported as a great source of fatty acids, protein, dietary fiber, minerals, and vitamins [14,15]. Furthermore, numerous studies identified the composition of flavonoids, carotenoids and anthocyanins, and other phenolic compounds in SPLs, such as phytoene, beta-carotene, lutein, antheraxanthin, violaxanthin, chlorophyll, caffeic acid, 3-caffeoylquinic acid, chlorogenic acid, 3,4-dicaffeoylquinic acid, 3,5-dicaffeoylquinic acid, 4,5-dicaffeoylquinic acid, and 3,4,5-tricaffeoylquinic acid [16,17,18,19]. SPL extracts and their phenolic compounds have been demonstrated in anticancer [20], antioxidant [17], anti-inflammatories [21], anti-obesity [22], anti-diabetic activities [23]. However, most previous studies were restricted to the single cultivar, have a deficiency of comparison with well-known anti-inflammation drugs, or there is a lack of systemic study on anti-inflammatory signaling pathways in various SPL cultivars.

Therefore, the objectives of the current study are to investigate and anti-inflammatory, antioxidant activities and contents of bioactive ingredients in different SPL cultivars, and their molecular mechanism to reduce LPS-induced inflammation in vitro.

## 2. Materials and Methods

### 2.1. Preparation of SPL Extracts

The sweetpotato accessions used in this study were obtained from the USDA national germplasm center (Plant Genetic Resources Conservation Unit). Sweetpotato roots were planted 2 inches deep and about 2 inches apart (density of 5 cm × 5 cm) in a greenhouse and field conditions in late February (greenhouse) to April (field) at the University of Arkansas at Pine Bluff’s (UAPB) Agricultural Research Farm, Pine Bluff, Arkansas, during 2018 and 2019. The slips from sweetpotato accessions were planted in sterilized pro-mix soil in 12 cm vinyl pots with proper tags at the greenhouse After two months, tips were harvested every 15 days. Chemical fertilizer (N: P: K = 8: 8: 8) was used at a rate of 500 pounds/acre, and compost was used at a rate of 8000 pounds/acre in volume. After each harvest, 150 pounds/acre of ammonium sulfate was applied as additional fertilizer. Soap water was applied once a week to control the aphids. Three replications were used for a single cultivar. The greenhouse harvested leaves were weighed, washed with running tap water, packed in polyethylene bags, labeled, and frozen at −81 °C. Samples were freeze-dried using the MillRock Technology Freeze Dryer (MD3053, Kingston, NY, USA) and ground to powder using a Hamilton Beach Coffee Grinder (80333, Southern Pines, NC, USA), weighted, and stored in polyethylene bags. To obtain a polyphenolic extract, 0.2 g of each sample was shaken with 5 mL of 70% ethanol for 2 h. The extracts were then vacuum filtered through Whatman 42 filter paper (Whatman^®^, Maidstone, UK), and rinsed with 100% ethanol. The extracts were adjusted to 10 mL with 100% ethanol using a volumetric flask and prepared SPL powder using a nitrogen drier. Finally, SPL powders dissolved in DMSO at a concentration of 50 mg/mL and used for experiments on antioxidant and anti-inflammation.

### 2.2. Chemicals

Fetal bovine serum (FBS), antibiotic-antimycotic, and Dulbecco’s modified Eagle’s medium (DMEM) were purchased from Thermo Fisher Scientific Co. (Waltham, MA, USA) 2,2-Diphenyl-1-picrylhydrazyl (DPPH), Folin–Ciocalteu’s phenol reagent, Trolox, gallic acid, and lipopolysaccharide from *E. coli* (LPS) were obtained from Sigma Aldrich Co. (St. Louis, MO, USA). Dexamethasone (C22H29FO5) was obtained from Sigma Aldrich Co. (purity > 97%) and used as an anti-inflammatory medicine in the present study. Chlorogenic acid (purity > 98%), 3,4-dicaffeoylquinic acid (purity > 98%), and 3,5-dicaffeoylquinic acid (purity > 98%) were bought from Cayman Chemical Co. (Ann Arbor, MI, USA). Western blot protein antibodies, such as anti-iNOS (sc-7271), anti-IKK-α (sc-7606), anti-IκB-α (sc-1643), anti-NF-κB (sc-8008), anti-p-IκB-α (sc-8404), anti-β-actin (sc-47778), and anti-mouse IgG-HRP secondary antibody (sc-516102) were ordered from Santa Cruz Biotechnology (Dallas, TX, USA), and anti-p-IKK-α (16A6), anti-COX-2 (D5H5), anti-Lamin B1 (D4Q4Z) and anti-rabbit IgG-HRP secondary antibody (7074) were purchased from Cell Signaling Technology (Danvers, MA, USA).

### 2.3. DPPH Radical Scavenging Activity

The free radical scavenging activity of extracts was measured by DPPH using an adapted method described by Akkari et al. [24]. A volume of 140 μL solution of DPPH in methanol (0.1 M) was added to 10 μL of each phenolic extract diluted 15× with methanol, incubated in a dark room for 30 min, then the absorbance was measured at 517 nm. The Radical scavenging activity was calculated using the following equation: Scavenging effect = [(A0 − A1)/A0] × 100, where A0 was the absorbance of the control and A1 was the absorbance in the presence of the sample against extract. DPPH radical scavenging activities were independently identified in replicate analysis (*n* = 6). Total antioxidant capacity was determined by fitting the scavenging effect into the linear regression line of a Trolox standard curve (31.25, 62.5, 125, 250, 500 μM).

### 2.4. Total Phenolic Contents Analysis

The amount of total phenolics in the extracts was determined using a modified version of the Folin-Ciocalteu assay described by Slinkard, and Singleton [25] using gallic acid as the reference standard (3.1, 6.25, 12.5, 25, 50, 100 mg gallic acid per liter, R2 = 0.99). The modification converted to method into a microassay using a microplate reader. Samples were diluted 15× with water. To complete the assay 20 µL of the sample, standard, or blank was placed in separate wells of a 96 well plate. To each well, 100 µL of 0.2 N Folin-Ciocalteu reagent and 80 µL of 0.7 M sodium carbonate was added. The plate was allowed to incubate for 2 h and samples were read at 760 nm. Total phenolic contents were independently identified in replicate analysis (*n* = 6). Results were expressed as milligrams of gallic acid equivalents per gram of freeze-dried powder.

### 2.5. High-Performance Liquid Chromatography (HPLC) Analysis

Phenolic compounds in SPL extracts were analyzed using a Shimadzu HPLC system (Shimadzu Co., Columbia, MD, USA). The system instruments were composed of a binary pump (LC-20AB), an auto sample injector (SIL-10AF), and a photodiode array detector (SPD-M20A). A YMC-Pack ODS-AM column (250 mm × 10 mm, 5 μm, YMC Co., Ltd.) was applied to detect chlorogenic acid and 3,4- and 3,5-dicaffeoylquinic acid at 326 nm wavelength. The column was maintained at ambient temperature. Mobile phase A was 0.2% formic acid in distilled water, and phase B was methanol (HPLC grade, Sigma Aldrich Co., St. Louis, MO, USA). The elution profile was 0–65 min, 5–75% B. The flow rate was 1 mL/min, and the injection volume was 50 μL. Phenolic compounds were independently demonstrated in at least triplicate.

Pigments in SPL extracts were also analyzed using a Shimadzu HPLC system (Shimadzu Co., Columbia, MD, USA). An analytical polymeric YMC C30 column (250 mm × 4.6 mm, 5 μm) was used for the separation of chlorophyll B, lutein, and chlorophyll A and β-carotene at room temperature. Mobile phases were A: 100% methanol and B: 100% dichloromethane. Initial conditions were 5% B, and then we increased the ratio to 10% B in 10 min, then to 20% B at 15 min, to 40% B at 30 min, and to 60% B at 53 min before returning to initial conditions. The flow rate was 1 mL/min, and the injection volume was 50 μL. Pigments were independently demonstrated in at least one duplicate.

### 2.6. Cell Culture

RAW 264.7 murine macrophage cells were purchased from ATCC (Manassas, VA, USA). Cells were maintained in DMEM supplemented with 200 mM of L-glutamine, 100 IU/mL, and 10% of FBS. Cells under 20 of passage numbers were used for NO, TNF-α, IL-1β, and PGE_2_ determinations.

### 2.7. 3-(4,5-Dimethylthiazol-2-yl)-5-(3-Carboxymethoxyphenyl)-2-(4-Sulfophenyl)2H-Tetra-Zolium (MTS) Assay

Cell viability was assessed by the Cell Titer MTS solution provided by Promega Co. (Madison, WI, USA). RAW 264.7 cells were seeded onto 96-well plates at a density of 1 × 10^4^ per well and incubated at a CO_2_ incubator (37 °C, 5% CO_2_) for 24 h. Cells were treated with 0.1% of DMSO in control, 50–500 μg/mL of SPL, and 1–20 μM of dexamethasone for 24 h. To quantify cell viability, 20 μL of MTS reagent was added to each well, and then incubated in a CO_2_ incubator for 2 h. Cell viability was determined by measuring the absorbance at 490 nm using a microplate reader (Synergy HT Multi-Mode Microplate Reader, BioTek Instruments, Inc., Winooski, VT, USA), and expressed as a percentage compared to control cells. Cell viability was independently identified in triplicate analysis (*n* = 9).

### 2.8. Nitric Oxide (NO) Determination

The effect of SPL extracts and dexamethasone on LPS-stimulated NO production was measured by using the Griess reagent commercial kit (Promega Co., Madison, WI, USA). RAW 264.7 cells were plated at a density of 1 × 10^4^/well in 96 well plates containing 100 μL of the medium and then incubated in a CO_2_ incubator overnight. Cells were pretreated with 0.1% of DMSO in the negative control (NC) and positive control (PC), 100–200 μg/mL of SPL extracts, and 20 μM of dexamethasone for 2 h and then stimulated with 100 ng/mL of LPS for 24 h. The culture supernatants were collected to measure nitrite contents. Equal volumes of Griess reagent and sample (50 μL) were incubated together at room temperature for 10 min (1:1 of 0.1% N-1-napthylethylendiamine dihydrochloride in water and 1% sulfanilamide in 5% phosphoric acid). Absorbance at 550 nm was measured using a microplate reader (BioTek Instruments, Inc. Winooski, VT, USA). Nitrite production, an indicator of NO synthesis, was calculated against a sodium nitrite standard curve. NO production levels were independently measured in triplicate analysis (*n* = 9).

### 2.9. Prostaglandin E_2_ (PGE_2_) Determination

The content of PGE_2_ was analyzed by Prostaglandin E_2_ monoclonal ELISA assay kit (Cayman Chemical Co., Ann Arbor, MI, USA). RAW 264.7 cells were seeded in 96 well plates containing 100 μL of DMEM at a concentration of 1 × 10^4^/well. After 24 h incubation, cells were maintained in a CO_2_ incubator for 2 h with 0.1% of DMSO in NC and PC, 200 μg/mL of SPL extracts, and 20 μM of dexamethasone, and then treated 100 ng/mL of LPS for 24 h. The culture mediums were mixed with fresh DMEM (1:30 dilution rate), and PGE_2_ contents in the culture medium were analyzed by the proposed protocol of the kit. The amounts of PGE_2_ in each sample and standards were measured at 405 nm by a microplate reader (BioTek Instruments, Inc. Winooski, VT, USA). PGE_2_ concentration of each sample was calculated using the equation obtained from the standard curve plot. PGE_2_ production levels were independently measured in replicate analysis (*n* = 8).

### 2.10. TNF-α and Interleukin-1β Determination

The TNF-α and IL-1β, pro-inflammatory cytokines were quantified by in vitro enzyme-linked immunosorbent ELISA assay kit (RayBiotech Co., Norcross, GA, USA). RAW 264.7 cells were seeded at a density of 1 × 10^4^/well in 96 well plates containing 100 μL of DMEM and maintained in a CO_2_ incubator for 24 h. Cells were pretreated with 0.1% of DMSO in NC and PC, 100–200 μg/mL of SPL extracts, and 20 μM of dexamethasone for 2 h and then stimulated with 100 ng/mL of LPS for 24 h. The culture supernatants were diluted 5 to 10 folds with a fresh DMEM medium to measure the content of cytokines. Cytokine proteins in the culture medium were attached to a microplate coated with an immobilized antibody for 2.5 h at room temperature. Next, TNF-α and IL-1β biotinylated antibodies were added to each plate for 1 h, and the plates were incubated with specific HRP-conjugated streptavidin for 1 h. After washing away unbounded antibodies, TMB substrate solution was added to each plate, and the degree of color developments in proportion to the amounts of cytokines was measured at 450 nm by a microplate reader (BioTek Instruments, Inc. Winooski, VT, USA).

### 2.11. Western Blotting Analysis

RAW 264.7 cells were spread into a 6-well plate at a concentration of 6 × 10^5^/well. After 24 h adjustment time in a CO_2_ incubator, SPL extracts (200 µg/mL) and dexamethasone (20 µM) were pretreated for 2 h, and then 100 ng/mL of LPS were treated for 1 h (IKK-α and IκB-α), 6 h (NF-κB) and 12 h (iNOS and COX-2). NC and PC cells were pretreated with 0.1% of DMSO for 2 h. The cells were lysed by RIPA cell lysis buffer (ThermoFisher Scientific Co., Waltham, MA, USA), and a BCA protein assay kit was used for the determination of protein concentration. Equal amounts of protein samples were electrophoresed in 12% of sodium dodecyl sulfate-polyacrylamide gel at 100 voltages. Loaded protein samples were transferred onto a polyvinylidene difluoride membrane (Bio-Rad Laboratories Inc., Hercules, CA, USA) at 110 voltages for 90 min. The protein transferred membranes were washed with 0.1% of T-TBS buffer (0.1% of tween 20 in Tri-Based Saline buffer), and then the protein-blocking process was performed using 5% of bovine calf albumin (BCA) in T-TBS buffer. The specific primary antibodies were treated to membranes at 4 °C for 12 h (dilution rate 1:1000), and secondary antibodies were attached to membranes at room temperature for 1 h (dilution rate 1:10,000). The protein bands on the membrane were detected using an enhanced chemiluminescence (ECL) kit in the darkroom. The quantification of the protein band was analyzed by Image Studio™ Lite software (LI-COR Inc., NE, USA) based on β-actin protein bands. Cytosol and nuclear NF-κB protein levels and total and phosphorylated IKK-α and IκB-α protein levels were quantified in duplicate.

### 2.12. Isolation of Nuclear and Cytosol Fraction

To measure nuclear translocated NF-κB proteins, nuclear and cytosol fractions were separated by NE-PER nuclear-cytoplasmic extraction kit (Thermo Fisher Scientific Co., Waltham, MA, USA) in RAW 264.7 cells. Briefly, after pretreatment of SPLs and dexamethasone for 2 h and treatment of LPS for 6 h, cells were lysed with cell extraction reagent I and II in ice for 20 min. Then, cytoplasmic fractions were isolated by microcentrifuge (16,000× *g*, 5 min). Nuclei pellet was extracted with nuclear extraction reagent in ice for 60 min. The nuclear fractions were also isolated by microcentrifuge (16,000× *g*, 5 min). Protein levels of fractions were quantified BCA protein assay kit, and NF-κB protein levels were investigated by Western blotting.

### 2.13. Statistical Analysis

The statistical analyses for inflammation studies were determined by one-way analysis of variance (ANOVA) with differences analyzed using Dunnett’s test. Statistical significance was expressed as * *p* < 0.05, ** *p* < 0.01, and *** *p* < 0.001. DPPH and total phenolics were statistically analyzed using Student’s t-test, confidence quantile 0.05 (SAS 9.4, SAS Institute Inc., Cary, NC, USA). The multivariant analysis was used to determine correlations between total phenolics and the DPPH assay.

## 3. Results

### 3.1. Effect of SPL Extracts in 29 Cultivars on Total Phenolic Contents and Antioxidant Activity

To investigate the antioxidant activity and total phenolic contents of SPL extracts, 29 cultivars harvested in 2018 and 2019 were analyzed by Folin–Ciocalteu and DPPH radical scavenging assay. In the 2018 SPL, total phenolic contents and DPPH radical scavenging activity showed 9.4–23.1 mg gallic acid equivalent/g and 36.6–190.0 mM of Trolox equivalent/g, respectively (Table 1). In the 2019 SPLs, total phenolic contents and DPPH radical scavenging activity demonstrated 9.7–17.6 mg gallic acid equivalent/g and 52.7–247.3 mM of Trolox equivalent/g, respectively (Table 1). Among 29 cultivars, SPL extracts #24, #20, #26, #34, #32, and #1 in 2018, and #1, #9, #3, #7, #10, and #38 in 2019 showed the highest total phenolic contents. SPL extracts #26, #24, #34, #20, #21, and #1 in 2018, and #1, #3, #9, #38, #11 and #15 in 2019 showed the highest antioxidant activities. These results indicate that although SPL extracts have a wide range of total phenolic content and antioxidant activity according to cultivars and harvest years, the higher phenolic contents in SPL extracts relate to antioxidant activity.

### 3.2. Phenolic and Pigment Composition of SPL Extracts

**Figure 1:**
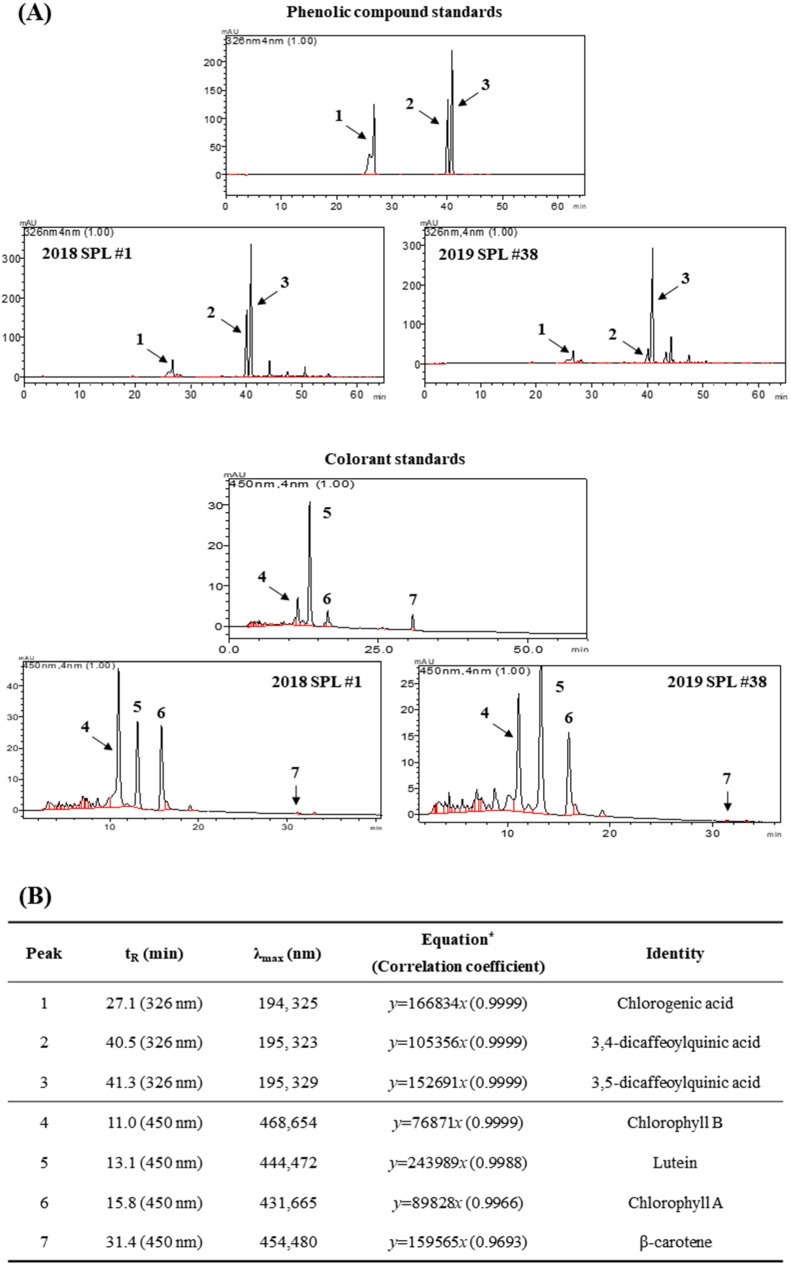
Chromatography of phenolic compounds and pigments in standards and SPL. High performance liquid chromatography (HPLC) was used for analysis of phenolic compounds and pigments in SPL at 326 nm and 450 nm of wavelength. (**A**) Chromatography peaks of phenolic compounds (peak #1, 2 and 3) and pigments (peak #4, 5, 6 and 7) in standards and sweetpotato leaves. (**B**) Peak assignments, retention times and wavelengths of maximum absorption for standard phenolic compounds. * y expresses the detection response (peak area), and x is the concentration for phenolic compounds. 1: Chlorogenic acid, 2: 3,4-dicaffeoylquinic acid, 3: 3–5-dicaffeoylquinic acid, 4: Chlorophyll B, 5: Lutein, 6: Chlorophyll A and 7: β-carotene.

Considering total phenolic content, antioxidant activity, and remained sample quantity, three kinds of SPL cultivars harvested in 2018 (#1, #20, and #32) and 2019 (#1, #3, and #38) were selected to proceed with further study. Phenolic and pigment contents in SPL extracts were measured using HPLC. Chlorogenic acid (ChA), 3,4-dicaffeoylquinic acid (3,4-diCQA), and 3,5-dicaffeoylquinic acid (3,5-diCQA) were investigated as major phenolics (Figure 1A,B), and chlorophyll B, lutein, chlorophyll A, and β-carotene were also identified as dominant colorant components indicating largest proportion in SPL extracts (Figure 1A,B). The contents of ChA, 3,4-diCQA, and 3,5-diCQA were demonstrated by 11.7–22.1, 16.3–59.9, and 50.9–72.7 µg/mg, respectively. In addition, 5.62–25.07 μg/mg of chlorophyll B, 2.34–5.15 μg/mg of lutein, 4.95–11.83 μg/mg of chlorophyll A, and 0.02–0.12 μg/mg of β-carotene were also identified in six kinds of SPL extracts (Table 2). These results suggest that various bioactive components are involved in the anti-inflammatory effect of SPL extracts.

### 3.3. Effect of SPL Extracts on Cell Viability and LPS-Induced Nitric Oxide (NO) and Prostaglandin E2 (PGE_2_) Production in RAW 264.7 Cells

**Figure 2:**
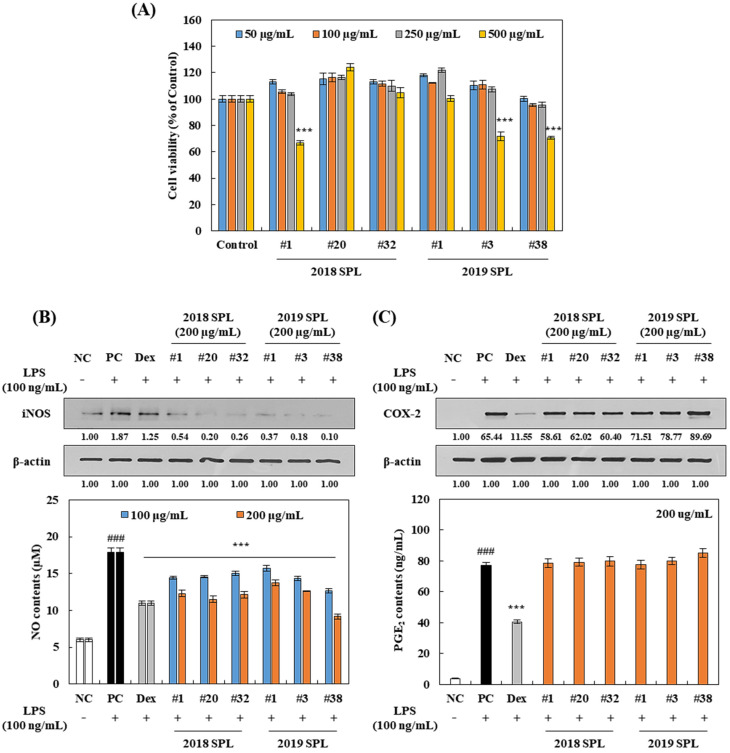
Effect of 2018 and 2019 SPL on LPS-induced NO and PGE_2_ production in RAW 264.7 cells. Cells were treated with 50–500 µg/mL of SPL for 24 h, and (**A**) cell viability was measured by MTS assay. (**B**) LPS-induced NO concentration was measured by Griess reagent system kit with NO standard, and iNOS expression was analyzed by Western blotting. (**C**) LPS-stimulated PGE_2_ contents were identified by Prostaglandin E2 ELISA Kit, and COX-2 expression was analyzed by Western blotting. RAW 264.7 cells were pretreated 100–200 µg/mL of SPL and 20 µM of dexamethasone (Dex) for 2 h, and then cells were incubated with 100 ng/mL of LPS for 24 h (NO and PGE_2_ contents analysis) and 12 h (Western blotting). Data are shown as mean ± SEM. Significant differences are compared with control at ### *p* < 0.01, and PC at *** *p* < 0.001 (*n* = 9, cell viability; *n* = 9, NO contents; *n* = 8, PGE_2_ contents).

To investigate the cytotoxicity of SPL on RAW 264.7 cells, an MTS assay was performed for six SPL extracts (Figure 2A). Treatment of SPL extracts (#1 in 2018 and #3 and #38 in 2019) at the concentrations of 500 μg/mL for 24 h significantly decreased RAW 264.7 cell viability (*p* < 0.001). Because there was no cytotoxicity until 250 μg/mL of SPL treatment, the further anti-inflammation study proceeded at a range of concentration 100–200 μg/mL.

RAW 264.7 cells were pretreated with 100–200 μg/mL of SPL extracts and stimulated with 100 ng/mL of LPS for 24 h to investigate the anti-inflammatory effects. Compared to LPS-untreated cells (negative control, NC), LPS-treated cells (positive control, PC) showed a significant increase of in NO and PGE_2_ contents in RAW 264.7 cells by 298% and 1983%, respectively (Figure 2B,C). Pretreatment of SPLs (100–200 μg/mL) in RAW 264.7 cells dose-dependently inhibited NO compared to PC by 12–48% (Figure 2B). However, PGE_2_ contents were not affected by pretreatment of SPL extracts in LPS-stimulated RAW 264.7 cells (Figure 2C). As a negative control, the treatment of dexamethasone (20 μM) markedly down-regulated NO and PGE_2_ contents in comparison with PC by 38% and 47%, respectively (Figure 2B,C).

Previous studies identified that iNOS and COX-2 proteins are the major inducers of NO and PGE_2_, respectively [8,9,10]. Western blot analysis revealed that LPS-treated cells significantly increased iNOS and COX-2 expression in RAW 264.7 cells by 87% and 6544%, respectively (Figure 2B,C). Similar to NO and PGE_2_ analysis data, pretreatment of SPL extracts sharply blocked LPS-induced iNOS protein expression by 32–94% compared to PC cells. However, there is no significant effect on COX-2 suppression in SPL-pretreated RAW 264.7 cells. Treatment of dexamethasone (20 μM) effectively blocked LPS-induced COX-2 expression compared to PC by 82%. These findings suggest that SPL extracts effectively inhibit LPS-induced NO production in RAW 264.7 cells without significant cytotoxicity.

### 3.4. Effects of SPL Extracts on Production of Pro-Inflammatory Cytokines in LPS-Stimulated RAW 264.7 Cells

**Figure 3:**
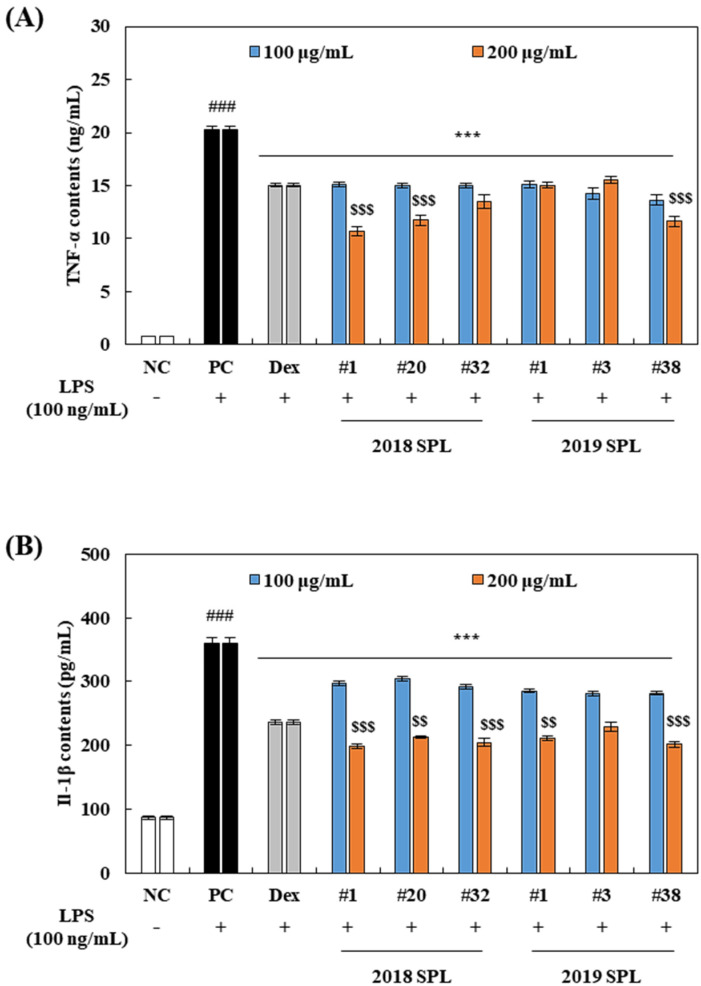
Effect of 2018 and 2019 SPL on production of TNF-α and IL-1β in RAW 264.7 cells. RAW 264.7 cells were pretreated 100–200 µg/mL of SPL and 20 µM of dexamethasone (Dex) for 2 h, and then cells were incubated with 100 ng/mL of LPS for 24 h. LPS-induced proinflammatory cytokines, (**A**) TNF-α and (**B**) IL-1β are measured by commercial kit with TNF-α and IL-1β standard. Data were calculated as mean ± SEM. Significant differences are compared with control at ### *p* < 0.01, PC at *** *p* < 0.001 and Dex at $$ *p* < 0.01, $$$ *p* < 0.001 (*n* = 5).

TNF-α and IL-1β, well-known inflammation-inducing cytokines, are closely related to the production of NO and induction of inflammation [8]. As shown in Figure 3A,B, the treatment of LPS (100 ng/mL) induced a dramatic increase in TNF-α and IL-1β production in RAW 264.7 cells. However, pretreatment of dexamethasone (20 μM) and SPL extracts (100 and 200 μg/mL) for 2 h significantly down-regulated TNF-α and IL-1β expressions compared to PC group cells (*p* < 0.001). Furthermore, pretreatment of some SPL extracts at a concentration of 200 μg/mL showed statistically lower TNF-α (2018: #1 and #20; 2019: #38) and IL-1β (2018: #1, #20, #32; 2019: #1 and #38) contents compared to dexamethasone-treated cells (*p* < 0.01 and *p* < 0.001) (Figure 3A,B). These data indicate that SPL extracts suppressed TNF-α and IL-1β production in LPS-stimulated RAW 264.7 cells.

### 3.5. Effects of SPL Extracts on the Regulation of NF-κB Signaling Pathway in LPS-Stimulated RAW 264.7 Cells

**Figure 4:**
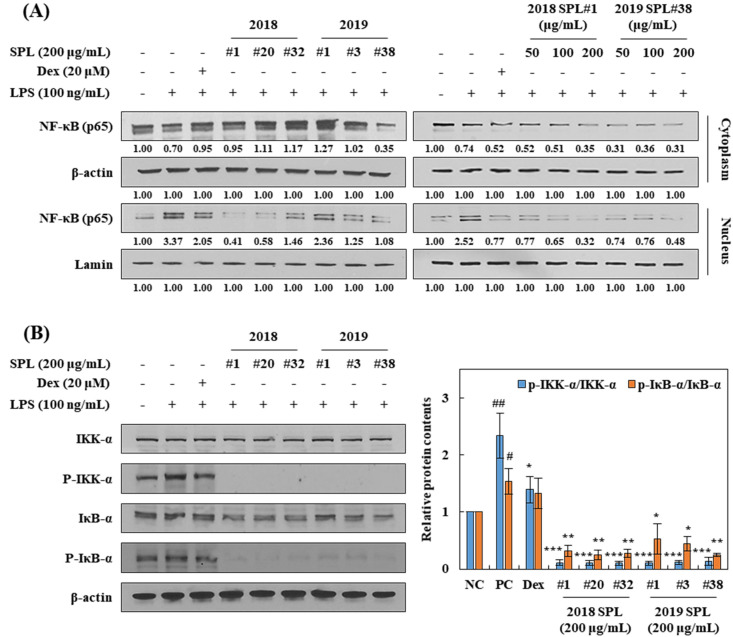
Effects of SPL extracts on nuclear factor (NF)-κB translocation in RAW 264.7 cells. Cells were pretreated with 200 μg/mL of SPL extracts and dexamethasone (20 μM) for 2 h. After pretreatment, cells were treated with LPS (100 ng/mL) for 6 h. To identify NF-κB protein expression in cytoplasm and nuclear, fractions were separated by NE-PER nuclear-cytoplasmic reagent kit. (**A**) Cytoplasm and nucleus NF-κB expression of 6 kind of SPL extracts was determined by Western blotting analysis according to cultivars and concentration. (**B**) IKK-α and IκB-α proteins were analyzed by Western blotting with 2 h pretreatment of SPL and dexamethasone, and 1 h treatment of LPS. Relative protein contents were quantified by Image J program. Data are presented as mean ± SEM. Significant differences are compared with control at # *p* < 0.05, ## *p* < 0.01, and PC at * *p* < 0.05, ** *p* < 0.01, *** *p* < 0.001 (*n* = 2).

NF-κB signaling pathway plays an important role in regulating the inflammatory response in macrophages [8]. To identify whether SPL inhibits translocation of NF-κB p65 protein from the cytoplasm to the nucleus, RAW 264.7 cells were fractionated into the cytoplasm and nucleus part, and the expression of NF-κB protein in each fraction was analyzed by Western blotting (Figure 4A,B). To compare with LPS-untreated cells, LPS-treated cells showed a significant increase in NF-κB protein translocation from cytoplasm to nucleus. However, the pretreatment of dexamethasone decreased the accumulation of NF-κB protein in the nucleus fraction. Pretreatment of SPL extracts also induced a dose-dependent decrease in NF-κB accumulation in the nucleus fraction of RAW 264.7 cells (Figure 4A,B). Moreover, RAW 264.7 cells were significantly down-regulated the LPS-induced expression of phosphorylated-IKK and phosphorylated-IκB proteins by pretreatment of SPL extracts and dexamethasone (Figure 4B). These data demonstrate that SPL inhibits LPS-induced inflammation in RAW 264.7 cells by blocking of NF-κB signaling pathway.

## 4. Discussion

Since the incidences of the inflammatory response and inflammation-related chronic disease are more closely involved in environmental factors than heredity [26,27], continuous management of inflammatory response is necessarily required in our routine. In this point of view, daily consumption of functional food materials that have various bioactivities such as antioxidant and anti-inflammation could be one of the effective ways to prevent chronic disease [28]. Sweetpotato leaves (SPL) have been showing biological activities such as anticancer [20], antioxidant [17], anti-inflammatory [21], anti-obesity [22], and anti-diabetic activities [23]. However, little has been known about the anti-inflammatory molecular mechanisms, phenolic composition, and antioxidant activity of SPL in various cultivars. In the present study, we compared radical scavenging activity and total phenolic content for 29 SPL cultivars harvested in 2018 and 2019. Although SPL harvested in 2019 showed more effective DPPH radical scavenging activity (52.7–247.3 mM of Trolox equivalent/g) than those in 2018 (36.6–190 mM of Trolox equivalent/g), the total phenolic content was slightly lower in 2019 SPLs (9.7–17.6 mg gallic acid equivalent/g) than the 2018 SPL (9.4–23.1 mg gallic acid equivalent/g). Previous studies have reported that various pigment ingredients including beta-carotene, lutein, and chlorophylls are also identified as major bioactive ingredients in SPL [16], suggesting that there is a synergistic antioxidant potential between specific phenolic compounds and pigment ingredients in SPL extracts. Moreover, many studies reported that the synthesis of phenolic compounds and/or bioactive substances in natural products can be highly affected by circumstantial factors including sunshine duration, temperature, soil, fertilizer, moisture, and harvest period [8,29]. These explain that the total phenolic contents and antioxidant activities on the same cultivar of SPL harvested in 2018 and 2019 can be different depending on environmental factors.

Numerous studies have tried to find novel biological activities of some nutrients and natural compounds in the viewpoint of value-added functional food [11,30,31]. Among them, phenolic compounds, secondary metabolites broadly distributed in plant sources, are majorly regarded as a potential candidate for the development of functional food and natural medicine for some chronic diseases, such as obesity, diabetes, cancer, inflammation, and hepatitis. Previous studies have also revealed that SPL, a major by-product of sweetpotato, has high contents of phenolic compounds, anthocyanins (purple SPL), chlorophylls, and carotenoids [16,17,18,19]. In the present study, we found three major phenolic compounds in SPL cultivars: chlorogenic acid (ChA, 11.7–22.1 µg/mg), 3,4-dicaffeoylquinic acid (3,4-diCQA, 16.3–59.9 µg/mg) and 3,5-dicaffeoylquinic acid (3,5-diCQA, 50.9–72.7 µg/mg) (Figure 1A and Table 2). The current findings are consistent with the study of Jang and Koh [18] and Islam et al. [19], demonstrating that 1.38–6.43 µg/mg of ChA, 1.01–11.53 µg/mg of 4,5-diCQA, 4.21–35.03 µg/mg of 3,5-diCQA, and 0.43–7.50 µg/mg of 3,4-diCQA are the leading phenolic components in SPL. Interestingly, SPL extracts showed various content ratios of ChA, 3,4-diCQA, and 3,5-diCQA. Based on these results, we further analyzed whether the ratio and content of these three major phenolic components are a leading cause majorly related to the anti-inflammatory effect of SPLs (Supplementary data). Compared to LPS-treated RAW 264.7 cells, pretreatment of ChA, 3,4-diCQA, and 3,5-diCQA for 2 h did not show a significant difference in NO production by single- and combined-treatment manners (Supplementary data). Moreover, the pigment components such as chlorophyll B (5.62–25.07 μg/mg), lutein (2.34–5.15 μg/mg), chlorophyll A (4.95–11.83 μg/mg), and β-carotene (0.02–0.12 μg/mg) were also identified in six kinds of SPL extracts by HPLC analysis (Table 2). Apart from these kinds of pigments, various studies have reported that SPL includes anthocyanins, lutein derivatives, pheophytins, and xanthine derivatives [16,17,18,19,32]. Collectively, these data indicate that SPL-induced anti-inflammatory effect is partly involved in the ratio and content of major phenolic compounds in SPLs such as ChA, 3,4-diCQA, and 3,5-diCQA; however, future studies are needed to reveal the relevance and synergistic effect of unknown phenolic compound peaks and the pigment components in the anti-inflammation effects of SPL.

Numerous reports have investigated that excessive production of reactive oxygen species and/or reactive nitrogen species plays an important role as a pro-inflammatory stimulator [33,34]. Furthermore, polyphenol-rich food materials have been reported as a great source of functional food for antioxidants and anti-inflammation [35]. Among the various kinds of pro-inflammatory factors, NO and PGE_2_ are the major inflammatory hallmarks that are activated under a variety of pathological conditions and immune responses [8,9,10]. To identify these inflammatory markers, RAW 264.7 cells were pretreated with 100–200 µg/mL of SPL and 20 µM of dexamethasone for 2 h, and then stimulated cells with 100 ng/mL of LPS treatment for 24 h. Results reported in the present study indicated that SPL effectively inhibited LPS-induced NO production in a dose-dependent manner (Figure 2B). Rather interestingly, we identified that SPLs did not affect the suppression of PGE_2_ production under LPS-stimulation (Figure 2C). In agreement with these data, iNOS protein was significantly down-regulated by SPL pretreatment, but the COX-2 expression degree was not changed in SPL-pretreated cells compared to LPS-treated positive control cells (Figure 2B,C). This suggests that the anti-inflammatory effect of SPL is majorly related to inhibition of the iNOS-nitric oxide pathway. The previous finding reported that 400–1000 µg/mL of SPL water extract significantly inhibited LPS-stimulated NO production in RAW 264.7 cells [21]. In addition, Zengin et al. [36] reported that 100–1000 µg/mL of SPL 60% methanol extract markedly suppressed LPS-induced NO and H_2_O_2_-induced PGE_2_ production in RAW 264.7 cells. Since H_2_O_2_-induced PGE_2_ production is more directly related to the antioxidant activity of SPL than LPS and the COX-2 signaling pathway involved case, the effect of SPL on PGE_2_ inhibition could be shown in only oxidative stressed RAW 264.7 cells, not the suppression of the COX-2 and NF-κB signaling pathways. On the other hand, previous studies did not suggest cytotoxicity data and comparison with a synthetic drug in inflammation data [21,36,37]. It indicates that reduction of the inflammatory marker by SPL could be caused by cytotoxicity of cells and they could not imply dose-efficacy of SPL compared to drug. In the present study, 2019 #38 SPL showed a stronger NO inhibition rate than dexamethasone (Figure 2B), and 2018 #1, #20 and 2019 #38 SPL significantly suppressed LPS-induced TNF-α and IL-1β levels compared with dexamethasone (Figure 3A,B). Therefore, the present data demonstrate that the 70% ethanol extracts of SPL indicate the anti-inflammatory effect against LPS stimulation at concentrations of 100–200 µg/mL without cytotoxicity.

The NF-κB signaling pathway plays a pivotal role as a mediator of the pro-inflammatory gene and cytokine production in innate immune cells [8]. Through the bacterial stimulation of TLR4, sequential signaling pathways such as IKKs, IκBα, and NF-κB translocation are activating, and this set of procedures finally leads to the production of pro-inflammatory cytokines including IL-1β, IL-1, -2, -6, -8, -12, -23, and TNF-α [8,9,10]. In the present study, pretreatment of six kinds of SPL extracts dose-dependently down-regulated TNF-α and IL-1β cytokine production (Figure 3A,B) and the translocation of NF-κB (p65) into the nucleus in LPS-treated RAW 264.7 cells (Figure 4A). Furthermore, ratios of p-IKK-α/IKK-α and p-IκBα/IκBα were significantly reduced by pretreatment of 200 µg/mL of SPL extracts in LPS-treated RAW 264.7 cells. These results showed a consistent tendency with our previous results, iNOS expression, and NO production (Figure 2B,C). Previous studies have reported that SPL extracts showed an inhibitory effect on the production of NO and PGE_2_, expression of iNOS, TNF-α, and IL-6 at the concentrations of 1–5 mg/mL in LPS- or H_2_O_2_ stimulated RAW 264.7 cells [21,36,37]. Although these studies suggested the possibility that SPL extracts had the anti-inflammation effect against LPS- or H_2_O_2_ stimulation, specific molecular mechanisms were not investigated. In the present study, our findings demonstrate that SPL extracts inhibit LPS-stimulated NO and pro-inflammatory cytokine productions through suppression of NF-κB signaling nuclear translocation and its upstream pathways, including p-IKK-α and p-IκBα. However, future investigations are required to confirm that species of pro-inflammatory cytokines such as IL-1, -2, -6, -8, -12, and -23 are affected by SPL 70% ethanol extracts.

## 5. Conclusions

This study identified that SPL effectively suppressed LPS-induced inflammation via inhibition of iNOS expression and the NF-κB signaling pathway in macrophages. Interestingly, LPS-induced COX-2 expression and PGE_2_ production were not affected by the treatment of SPL extracts. Although further studies are required to identify the relevance of major bioactive compounds on the anti-inflammation effect of SPL, the present findings suggest that SPL cultivars containing high contents of phenolics could be used as antioxidants and anti-inflammatory functional food ingredients.

## Figures and Tables

**Table 1:** Total polyphenols and antioxidant capacity of sweet potato leaves (SPL).

SPLs	Total Polyphenols (mg Gallic Acid Equiv/g)		Antioxidant Capacity (mmol Trolox Equiv/g)	
	2018	2019	2018	2019
**#1**	15.5 ± 0.4 ^fg^	17.6 ± 0.3 ^a^	117.8 ± 26.7 ^bcd^	247.3 ± 18.0 ^a^
**#3**	12.2 ± 0.1 ^m^	14.7 ± 0.3 ^bc^	76.9 ± 5.8 ^defghi^	136.7 ± 11.5 ^b^
**#5**	14.0 ± 0.2 ^ij^	-	109.7 ± 5.0 ^bcde^	-
**#7**	13.2 ± 0.2 ^l^	14.3 ± 0.2 ^bcd^	53.7 ± 5.1 ^fhi^	95.5 ± 8.0 ^efgh^
**#8**	11.5 ± 0.2 ^no^	-	75.0 ± 2.8 ^defghi^	-
**#9**	11.0 ± 0.2 ^op^	14.8 ± 0.3 ^b^	36.6 ± 10.0 ^i^	117.4 ± 8.1 ^c^
**#10**	9.7 ± 0.2 ^qr^	14.2 ± 0.5 ^cd^	39.4 ± 1.4 ^i^	103.4 ± 9.6 ^def^
**#11**	11.4 ± 0.1 ^nop^	13.8 ± 0.5 ^de^	51.3 ± 11.6 ^hi^	106.4 ± 13.2 ^cde^
**#15**	9.4 ± 0.1 ^r^	12.7 ± 0.5 ^gh^	37.5 ± 5.0 ^i^	105.9 ± 8.4 ^cde^
**#16**	14.6 ± 0.1 ^hi^	12.2 ± 0.2 ^hij^	79.3 ± 8.9 ^defghi^	96.6 ± 10.5 ^efgh^
**#18**	15.5 ± 0.1 ^fg^	13.4 ± 0.2 ^ef^	68.9 ± 5.0 ^efghi^	103.8 ± 9.0 ^def^
**#19**	14.0 ± 0.0 ^ij^	12.5 ± 0.4 ^hi^	63.6 ± 17.5 ^fghi^	100.3 ± 9.9 ^defg^
**#20**	19.1 ± 0.1 ^c^	-	149.1 ± 12.8 ^ab^	-
**#21**	17.8 ± 0.2 ^d^	11.9 ± 0.3 ^j^	146.7 ± 13.8 ^abc^	81.4 ± 14.2 ^ijkl^
**#23**	15.8 ± 0.1 ^f^	12.1 ± 0.2 ^ij^	95.9 ± 21.1 ^defg^	87.3 ± 15.4 ^hijk^
**#24**	23.1 ± 0.2 ^a^	9.7 ± 0.7 ^m^	181.4 ± 33.4 ^a^	52.7 ± 6.8 ^o^
**#26**	19.7 ± 0.2 ^b^	10.9 ± 0.9 ^l^	190.0 ± 16.2 ^a^	75.4 ± 11.2 ^klm^
**#28**	-	12.7 ± 0.5 ^gh^	-	90.5 ± 14.1 ^ghij^
**#32**	16.5 ± 0.0 ^e^	13.0 ± 0.4 ^fg^	105.9 ± 9.5 ^bcdef^	86.3 ± 9.8 ^hijkl^
**#34**	19.1 ± 0.1 ^c^	13.1 ± 0.6 ^fg^	161.9 ± 23.5 ^a^	92.4 ± 8.2 ^fghi^
**#35**	13.9 ± 0.1 ^jk^	11.2 ± 0.4 ^kl^	103.5 ± 15.0 ^cdef^	61.7 ± 13.8 ^no^
**#36**	9.9 ± 0.1 ^q^	12.5 ± 0.8 ^hi^	36.6 ± 2.8 ^i^	74.1 ± 11.8 ^mn^
**#37**	12.3 ± 0.1 ^n^	-	68.9 ± 7.8 ^efghi^	-
**#38**	15.4 ± 0.2 ^fg^	14.1 ± 0.5 ^d^	97.4 ± 8.6 ^def^	110.8 ± 10.0 ^cd^
**#39**	15.0 ± 0.1 ^gh^	11.8 ± 0.3 ^i^	92.6 ± 10.1 ^defgh^	77.9 ± 10.8 ^jkl^
**#40**	13.5 ± 0.1 ^kl^	13.3 ± 0.5 ^ef^	73.1 ± 10.9 ^efghi^	89.5 ± 8.3 ^ghij^
**#41**	11.9 ± 0.1 ^mn^	-	73.1 ± 6.7 ^efghi^	-
**#44**	-	11.7 ± 0.5 ^jk^	-	75.2 ± 9.4 ^klm^
**#45**	10.9 ± 0.2 ^p^	10.1 ± 0.4 ^m^	49.4 ± 9.3 ^hi^	63.7 ± 9.0 ^mno^

Data expressed as a range of data (mean ± SD) (*n* = 6). Values within columns with different letters are significantly different (*p* < 0.05). Equiv = equivalent; - = no sample.

**Table 2:** Contents of phenolic compounds and pigments in sweetpotato leaves (SPL).

Peak No./Compound		SPL (µg/mg)					
		2018 #1	2018 #20	2018 #32	2019 #1	2019 #3	2019 #38
Phenolics	**(1) Chlorogenic acid**	16.9 ± 0.5 ^bc^	11.9 ± 0.9 ^d^	22.1 ± 1.8 ^a^	20.2 ± 2.6 ^ab^	11.7 ± 0.6 ^d^	15.1 ± 0.7 ^cd^
	**(2) 3,4-dicaffeoylquinic acid**	59.5 ± 1.3 ^a^	57.4 ± 4.8 ^a^	52.6 ± 3.7 ^a^	59.9 ± 7.2 ^a^	30.3 ± 1.4 ^b^	16.3 ± 0.7 ^c^
	**(3) 3,5-dicaffeoylquinic acid**	72.7 ± 3.6 ^a^	50.9 ± 4.3 ^b^	69.3 ± 7.7 ^a^	69.6 ± 8.2 ^a^	63.5 ± 3.4 ^ab^	71.5 ± 5.9 ^a^
Pigments	**(4) Chlorophyll B**	9.3 ± 4.2 ^a^	6.1 ± 2.5 ^a^	12.3 ± 1.6 ^a^	8.8 ± 2.0 ^a^	9.2 ± 0.6 ^a^	8.2 ± 1.3 ^a^
	**(5) Lutein**	2.4 ± 0.7 ^b^	1.9 ± 0.5 ^b^	4.9 ± 0.9 ^a^	3.9 ± 1.0 ^ab^	3.7 ± 0.1 ^ab^	2.4 ± 0.8 ^b^
	**(6) Chlorophyll A**	3.5 ± 2.0 ^a^	2.7 ± 0.9 ^a^	3.9 ± 1.2 ^a^	4.3 ± 0.5 ^a^	3.3 ± 1.0 ^a^	3.9 ± 1.1 ^a^
	**(7) β-carotene**	0.1<	0.1<	0.1<	0.1	0.1	0.1<

Data expressed as mean ± SD (*n* = 3 for phenolics; *n* = 2 for pigments). Values within columns with different letters are significantly different (*p* < 0.05).

## Data Availability

Not Applicable.

## Supplementary Materials

The following are available online at https://www.mdpi.com/article/10.3390/foods10092051/s1, Supplementary data: Effects of single-, co- and triple-treatment of phenolic compounds on LPS-induced NO production in RAW 264.7 cells.

## References

[1] Zeyda M., Stulnig T.M. (2009). Obesity, inflammation, and insulin resistance—A mini-review. Gerontology.

[2] Sica A., Allavena P., Mantovani A. (2008). Cancer related inflammation: The macrophage connection. Cancer Lett..

[3] Barin J.G., Rose N.R., Cihakova D. (2012). Macrophage diversity in cardiac inflammation: A review. Immunobiology.

[4] Heppner F.L., Ransohoff R.M., Bacher B. (2015). Immune attack: The role of inflammation in Alzheimer disease. Nat. Rev. Neurosci..

[5] Merad M., Martin J.C. (2020). Pathological inflammation in patients with COVID-19: A key role for monocytes and macrophages. Nat. Rev. Immunol..

[6] Herold S., Mayer K., Lohmeyer J. (2011). Acute lung injury: How macrophages Orchestrate resolution of inflammation and tissue repair. Front. Immunol..

[7] Somasundaram V., Basudhar D., Bharadwaj G., No J.H., Ridnour L.A., Cheng R.Y.S., Fujita M., Thomas D.D., Anderson S.K., McVicar D.W. (2019). Molecular mechanisms of nitric oxide in cancer progression, signal transduction, and metabolism. Antioxid. Redox Signal..

[8] Liu T., Zhang L., Joo D., Sun S.C. (2017). NF-κB signaling in inflammation. Signal Transduct. Target. Ther..

[9] Thanos D., Maniatis T. (1995). NF-κB: A lesson in family values. Cell.

[10] Sun S.C. (2011). Non-canonical NF-κB signaling pathway. Cell Res..

[11] Caleja C., Ribeiro A., Barreiro M.F., Ferreira I.C.F.R. (2017). Phenolic compounds as nutraceuticals or functional food ingredients. Curr. Pharm. Des..

[12] Neela S., Fanta S.W. (2019). Review on nutritional composition of orange-fleshed sweet potato and its role in management of vitamin A deficiency. Food Sci. Nutr..

[13] Ayeleso T.B., Ramachela K., Mukwevho E. (2016). A review of therapeutic potentials of sweet potato: Pharmacological activities and influence of the cultivar. Trop. J. Pharm. Res..

[14] Johnson M., Pace R.D. (2010). Sweet potato leaves: Properties and synergistic interactions that promote health and prevent disease. Nutr. Rev..

[15] Sun H., Mu T., Xi L., Zhang M., Chen J. (2014). Sweet potato (*Ipomoea batatas* L.) leaves as nutritional and functional foods. Food Chem..

[16] Drapal M., Rossel G., Heider B., Fraser P.D. (2019). Metabolic diversity in sweet potato (*Ipomoea batatas*, L.) leaves and storage roots. Hortic. Res..

[17] Fu Z., Tu Z., Zhang L., Wang H., Wen Q., Huang T. (2016). Antioxidant activities and polyphenols of sweet potato (*Ipomoea batatas* L.) leaves extracted with solvents of various polarities. Food Biosci..

[18] Jang Y., Koh E. (2018). Antioxidant content and activity in leaves and petioles of six sweet potato (*Ipomoea batatas* L.) and antioxidant properties of Blanched leaves. Food Sci. Biotechnol..

[19] Islam M.S., Yoshimoto M., Yahara S., Okuno S., Ishiguro K., Yamakawa O. (2002). Identification and characterization of foliar polyphenolic composition in sweetpotato (*Ipomoea Batatas* L.) genotypes. J. Agric. Food Chem..

[20] Rie K., Adachi M., Yamakawa O., Yoshimoto M. (2007). Growth suppression of human cancer cells by polyphenolics from sweetpotato (*Ipomoea batatas* L.) leaves. J. Agric. Food Chem..

[21] Huang M.H., Chu H.L., Juang L.J., Wang B.S. (2010). Inhibitory effects of sweet potato leaves on nitric oxide production and protein nitration. Food Chem..

[22] Kurata R., Kobayashi T., Ishii T., Niimi H., Niisaka S., Kubo M., Kishimoto M. (2017). Influence of sweet potato (Ipomoea batatas L.) leaf consumption on rat lipid metabolism. Food Sci. Technol. Res..

[23] Nagamine R., Ueno S., Tsubata M., Yamaguchi K., Takagaki K., Hira T., Hara H., Tsuda T. (2014). Dietary sweet potato (Ipomoea Batatas L.) leaf extract attenuates hyperglycaemia by enhancing the secretion of glucagon-like peptide-1 (GLP-1). Food Funct..

[24] Akkari H., Hajaji S., B’chir F., Rekik M., Gharbi M. (2016). Correlation of polyphenolic content with radical-scavenging capacity and anthelmintic effects of Rubus ulmifolius (Rosaceae) against Haemonchus contortus. Vet. Parasitol..

[25] Slinkard K., Singleton V.L. (1977). Total phenol analysis: Automation and comparison with manual methods. Am. J. Enol. Vitic..

[26] Han D.Y., Fraser A.G., Dryland P., Ferguson L.R. (2010). Environmental factors in the development of chronic inflammation: A case-control study on risk factors for Crohn’s disease within New Zealand. Mutat. Res..

[27] Kelishadi R., Mirghaffari N., Poursafa P., Gidding S.S. (2009). Lifestyle and environmental factors associated with inflammation, oxidative stress and insulin resistance in children. Atherosclerosis.

[28] Serino A., Salazar G. (2019). Protective role of polyphenols against vascular inflammation, aging and cardiovascular disease. Nutrients.

[29] Penuelas J., Llusia J. (1997). Effects of carbon dioxide, water supply, and seasonality on terpene content and emission by Rosmarinus officinalis. J. Chem. Ecol..

[30] Santos-Buelga C., Gonzalez-Paramas A.M., Oludemi T., Ayuda-Duran B., Gonzalez-Manzano S. (2019). Plant phenolics as functional food ingredients. Adv. Food Nutr. Res..

[31] Maqsood S., Adiamo O., Ahmad M., Mudgil P. (2020). Bioactive compounds from date fruit and seed as potential nutraceutical and functional food ingredients. Food Chem..

[32] Chen B.H., Chen Y.Y. (1993). Stability of chlorophylls and carotenoids in sweet potato leaves during microwave cooking. J. Agric. Food Chem..

[33] Hussain T., Tan B., Yin Y., Blachier F., Tossou M.C., Rahu N. (2016). Oxidative stress and inflammation: What polyphenols can do for us?. Oxidative Med. Cell. Longev..

[34] Arulselvan P., Fard M.T., Tan W.S., Gothai S., Fakurazi S., Norhaizan M.E., Kumar S.S. (2016). Role of antioxidants and natural products in inflammation. Oxidative Med. Cell. Longev..

[35] Cory H., Passarelli S., Szeto J., Tamez M., Mattei J. (2018). The role of polyphenols in human health and food systems: A mini-review. Front. Nutr..

[36] Zengin G., Locatelli M., Stefanucci A., Macedonio G., Novellino E., Mirzaie S., Dvorascko S., Carradori S., Brunetti L., Orlando G. (2017). Chemical characterization, antioxidant properties, anti-inflammatory activity, and enzyme inhibition of Ipomoea batatas L. leaf extracts. Int. J. Food Prop..

[37] Sugata M., Lin C.Y., Shih Y.C. (2015). Anti-inflammatory and anticancer activities of Taiwanese purple-fleshed sweet potatoes (*Ipomoea batatas* L. Lam) extracts. BioMed Res. Int..

