# Supplementary material for: Sweetpotato Leaves Inhibit Lipopolysaccharide-Induced Inflammation in RAW 264.7 Macrophages via Suppression of NF-κB Signaling Pathway
Source: Foods. 2021 Aug 31;10(9):2051. doi: 10.3390/foods10092051 (PMC8464942; doi:10.3390/foods10092051)
Supplement: Supplementary file 1 [file foods-10-02051-s001.zip › foods-1338961-supplementary.pdf]

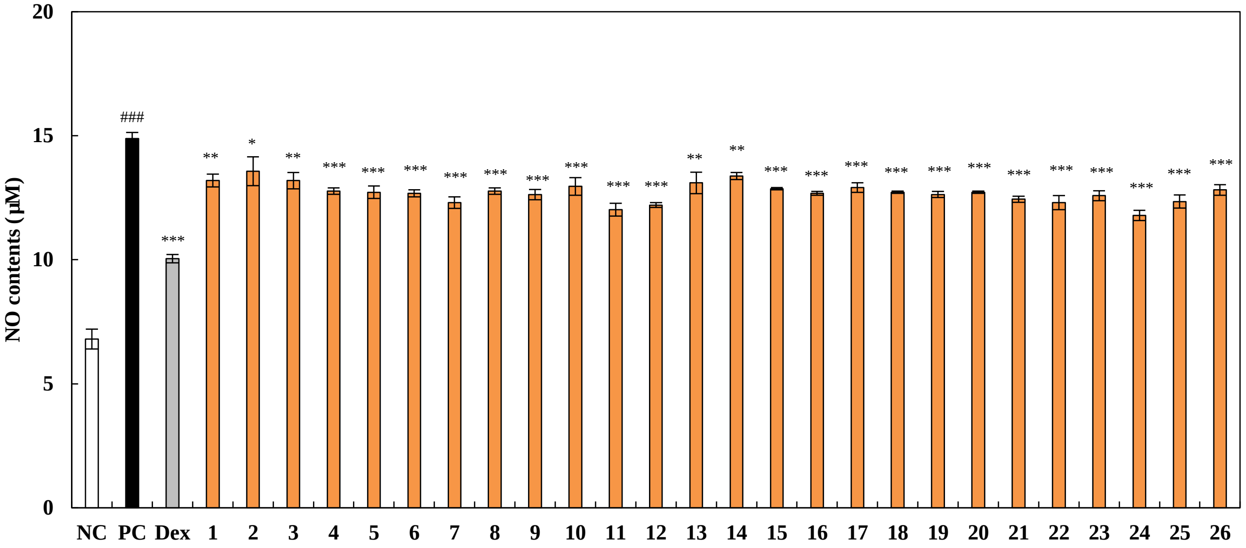

Supplementary data. Effects of single-, co- and triple-treatment of phenolic compounds on LPS-induced NO production in RAW 264.7 cells. RAW 264.7 cells were pretreated 12-84 μg/mL of chlorogenic acid, 3,4-dicaffeoylquinic acid and 3,5-dicaffeoylquinic acid and 20 μM of dexamethasone for 2 h, and then cells were incubated with 100 ng/mL of LPS for 24 h. LPS-induced NO concentration was measured by Griess reagent system kit with NO standard. Significant differences were compared with control at ###P<0.001, and PC at *P<0.05, **P<0.01 and ***P<0.001 (n = 3).

1. chlorogenic acid (12 μg/mL)
2. chlorogenic acid (24 μg/mL)
3. 3,4-dicaffeoylquinic acid (36 μg/mL)
4. 3,4-dicaffeoylquinic acid (72 μg/mL)
5. 3,5-dicaffeoylquinic acid (42 μg/mL)
6. 3,5-dicaffeoylquinic acid (84 μg/mL)
7. chlorogenic acid (12 μg/mL) + 3,4-dicaffeoylquinic acid (36 μg/mL)
8. chlorogenic acid (12 μg/mL) + 3,4-dicaffeoylquinic acid (72 μg/mL)
9. chlorogenic acid (24 μg/mL) + 3,4-dicaffeoylquinic acid (36 μg/mL)
10. chlorogenic acid (24 μg/mL) + 3,4-dicaffeoylquinic acid (72 μg/mL)
11. 3,4-dicaffeoylquinic acid (36 μg/mL) + 3,5-dicaffeoylquinic acid (42 μg/mL)
12. 3,4-dicaffeoylquinic acid (36 μg/mL) + 3,5-dicaffeoylquinic acid (84 μg/mL)
13. 3,4-dicaffeoylquinic acid (72 μg/mL) + 3,5-dicaffeoylquinic acid (42 μg/mL)
14. 3,4-dicaffeoylquinic acid (72 μg/mL) + 3,5-dicaffeoylquinic acid (84 μg/mL)
15. chlorogenic acid (12 μg/mL) + 3,5-dicaffeoylquinic acid (42 μg/mL)
16. chlorogenic acid (12 μg/mL) + 3,5-dicaffeoylquinic acid (84 μg/mL)
17. chlorogenic acid (24 μg/mL) + 3,5-dicaffeoylquinic acid (42 μg/mL)
18. chlorogenic acid (24 μg/mL) + 3,5-dicaffeoylquinic acid (84 μg/mL)
19. chlorogenic acid (12 μg/mL) + 3,4-dicaffeoylquinic acid (36 μg/mL) + 3,5-dicaffeoylquinic acid (42 μg/mL)
20. chlorogenic acid (12 μg/mL) + 3,4-dicaffeoylquinic acid (36 μg/mL) + 3,5-dicaffeoylquinic acid (84 μg/mL)
21. chlorogenic acid (12 μg/mL) + 3,4-dicaffeoylquinic acid (72 μg/mL) + 3,5-dicaffeoylquinic acid (42 μg/mL)
22. chlorogenic acid (12 μg/mL) + 3,4-dicaffeoylquinic acid (72 μg/mL) + 3,5-dicaffeoylquinic acid (84 μg/mL)
23. chlorogenic acid (24 μg/mL) + 3,4-dicaffeoylquinic acid (36 μg/mL) + 3,5-dicaffeoylquinic acid (42 μg/mL)
24. chlorogenic acid (24 μg/mL) + 3,4-dicaffeoylquinic acid (36 μg/mL) + 3,5-dicaffeoylquinic acid (84 μg/mL)
25. chlorogenic acid (24 μg/mL) + 3,4-dicaffeoylquinic acid (72 μg/mL) + 3,5-dicaffeoylquinic acid (42 μg/mL)
26. chlorogenic acid (24 μg/mL) + 3,4-dicaffeoylquinic acid (72 μg/mL) + 3,5-dicaffeoylquinic acid (84 μg/mL)
